# Effects of Combined Use of Ultrasonic Bone Scalpel and Hemostatic Matrix on Perioperative Blood Loss and Surgical Duration in Degenerative Thoracolumbar Spine Surgery

**DOI:** 10.1155/2019/6286258

**Published:** 2019-05-19

**Authors:** Hou-Tsung Chen, Chieh-Cheng Hsu, Meng-Lin Lu, Sung-Hsiung Chen, Jing-Miao Chen, Re-Wen Wu

**Affiliations:** ^1^Department of Orthopaedic Surgery, Kaohsiung Chang Gung Memorial Hospital, Kaohsiung, Taiwan; ^2^Graduate Institute of Clinical Medical Sciences, Chang Gung University, Taiwan

## Abstract

How to decrease intraoperative bleeding, shorten surgical time, and increase safety in spinal surgery is an important issue. Ultrasonic bone removers and FloSeal have been proven to increase safety, reduce the surgical duration, and decrease intraoperative bleeding in skull base surgery. Therefore, we aimed to compare the surgical duration, blood loss, and complications during spinal surgery with or without the use of FloSeal and an ultrasonic bone scalpel. Therefore, we retrospectively reviewed 293 patients who underwent thoracolumbar spinal surgery with decompression and instrumented fusion performed by a single surgeon. We divided these patients into three groups, including nonuse of FloSeal nor a bone scalpel (group A), use of FloSeal only (group B), and use of FloSeal and a bone scalpel (group C) intraoperatively after pairing in terms of age, sex, and surgical level. The surgical duration, blood loss, and occurrence of complications were all recorded. The mean surgical duration in group A was 160 mins, in group B it was 167 mins, and in group C it was 134 mins. The mean blood loss was 700 ml in group A, 682 ml in group B, and 383 ml in group C. Six patients sustained intraoperative dura injuries in total, 3 in group A, 2 in group B, and 1 in group C. No postoperative neurologic defects or occurrences of hematoma were recorded. According to our results, we concluded that combined use of FloSeal and bone scalpels is recommended during primary thoracolumbar spinal surgery to reduce the intraoperative blood loss and shorten the surgical duration.

## 1. Introduction

In light of increasing numbers of spinal surgeries and an increasing age of patients undergoing this surgery in recent decades, improving surgical safety and avoiding complications in spinal surgery are paramount. One of the most critical steps in thoracolumbar degenerative spinal surgery is the decompressive procedure, because it carries increased risks of nerve or dura injury, or unstoppable bleeding, and also requires a significant amount of time to perform. Therefore, a number of instruments have been developed to render the decompressive procedure safer and quicker, such as ultrasonic devices.

Traditionally, a Kerrison punch, an osteotome, and a high-speed drill were frequently used to perform the decompressive procedure, each of which differs in terms of the risk profile. The Kerrison punch is a classic device used for over 100 years in spinal surgery, with the benefits of availability of variable sizes, excellent cutting properties, and a low cost, but a long surgical duration is necessary if a multilevel-wide laminectomy is required. High-speed drills and ultrasonic devices have been frequently used for bone removal in spinal surgery recently owing to their similar techniques; however, close attention is needed while performing high-speed drilling to reduce the risk of thermal injury to bone and surrounding soft tissue and decrease the requirement for autologous bone grafts and incidental durotomies [1]. On the other hand, ultrasonic bone scalpel devices have been used in skull base surgery safely for years and were introduced into spinal surgery in recent years [2, 3]. Many studies have concluded that the ultrasonic bone scalpel is a safe tool that performs as desired when used as a bone-cutting device to facilitate osteotomies and offers greater bone-cutting precision and lessens damage to surrounding tissue, in addition to reducing blood loss [2, 4, 5].

Intraoperative bleeding is an issue in spinal surgery, in particular, during the decompressive procedure; it not only limits the surgical field, increasing the risks of spinal cord or nerve root injury, but also lengthens the surgical duration. Many studies have reported significant intraoperative blood loss to be associated with a greater incidence of morbidity and a prolonged length of hospital stay [6–8]. Numerous products, including unipolar or bipolar cautery, bone wax, antifibrinolytic agents, gelatin sponge (Gelfoam), oxidized regenerated cellulose (Surgicel), gelatine matrix (FloSeal), thrombin, and fibrin glue (Tisseel), are currently used to control perioperative bleeding and thereby shorten the surgical duration and increase safety.

Many studies have analyzed the efficacy, safety, and cost-effectiveness of the use of ultrasonic devices in spinal surgery and separately employing FloSeal to reduce the intraoperative blood loss in spinal surgery [2, 9–11], but no study has discussed the results of their combined use in primary thoracolumbar spine surgery. The aim of this study was to compare the surgical duration, intraoperative blood loss, and incidence of postoperative complications among three groups of patients who underwent primary thoracolumbar spinal surgery using neither an ultrasonic bone scalpel nor FloSeal, using FloSeal only, and using both an ultrasonic bone scalpel and FloSeal.

## 2. Materials and Methods

### 2.1. Patients

The records of patients from November 2011 to June 2016 who underwent thoracolumbar spinal surgery with decompression and instrumented fusion performed by a single surgeon were retrospectively reviewed. The study was conducted with a waiver of patient consent and was approved by the Institutional Review Board of our hospital (IRB201700086B0). The inclusion criteria for the retrospective analysis consisted of (1) patients aged from 25 to 90 years who underwent primary thoracolumbar surgery with wide decompression, pedicle screw instrumentation, and posterolateral fusion; (2) surgical levels ≥ 2 and ≤ 6; (3) use of neither hemostatic gel matrix FloSeal (Baxter Healthcare, Deerfield, IL, USA; 5 ml/pack) nor an ultrasonic bone scalpel (Misonix MXB-S1, Farmingdale, NY, USA), use of FloSeal only, or use of both, while the exclusion criteria consisted of revision thoracolumbar surgery, cervical spine surgery, spinal surgery with decompression only, preop coagulopathy, surgical levels ≥ 7, spinal tumor or infectious spondylitis, and preop neurologic deficits. Initially, 389 patients were considered for inclusion in the analysis; then, 96 patients were excluded for the following reasons: 62 patients with pure decompression surgery only, 4 patients with surgical levels ≥ 7, 9 patients with cervical surgery, and 21 patients who underwent revision spinal surgery. Finally, 293 patients in total were included in this study.

We divided these patients into three groups based on the surgical materials used during spinal surgery. In total, 96 patients, with a mean age of 65.3 ± 7.1 years, 42 men and 54 women, were classified into group A, in whom neither FloSeal nor a bone scalpel was employed during spinal surgery. In group B, there were 96 patients in total, with a mean age of 68.7 ± 6.3 years, 24 men and 72 women, in whom FloSeal was employed to complete the spinal surgery. Finally, group C included 101 patients with a mean age of 65.2 ± 6.5 years, 31 men and 70 women, in whom both FloSeal and an ultrasonic bone scalpel were employed during spinal surgery. The number of surgical levels has a direct impact on intraoperative blood loss and surgical duration; therefore, we further subclassified each group into short-segment or long-segment surgery based on the surgical levels, short-segment surgery being defined as surgical levels ≤ 3 and long-segment surgery as surgical levels ≥ 4 (Figure 2). The total intraoperative blood loss and surgical duration in each group, and in the short- and long-segment subgroups, were recorded and analyzed. The gender distribution, number of patients, patient age, decompression level, intraoperative assistance method, and number of cases in which FloSeal was used are presented in Table 1. The surgical duration, blood loss, and occurrence of complications were all recorded and are presented in Table 2.

### 2.2. Surgical Technique

**Figure 1:**
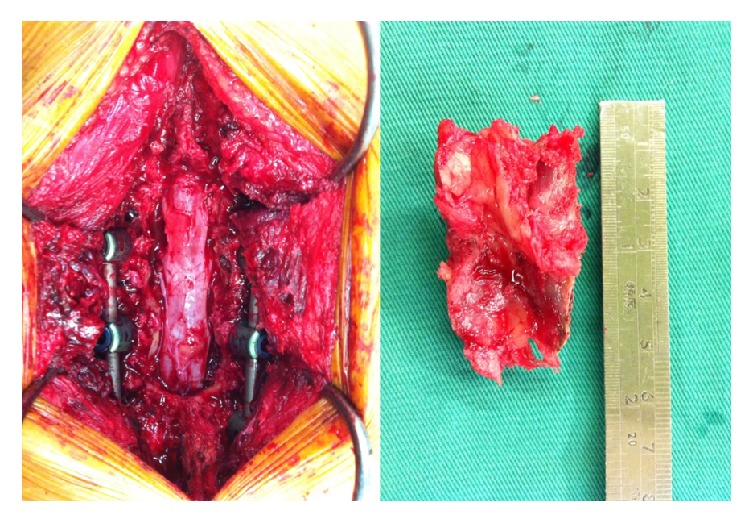
Decompression procedure with wide laminectomy by using ultrasonic bone scalpel in thoracolumbar spine surgery.

**Figure 2:**
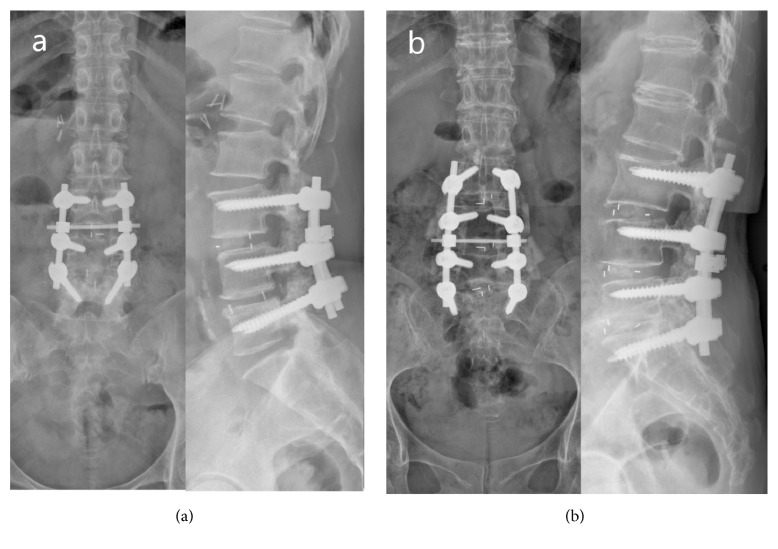
Both postoperative X-rays showed wide laminectomy decompressive procedure in degenerative thoracolumbar spine surgery (a, b). (a) 57 y/o female, degenerative spondylolisthesis with spinal stenosis of L345, who underwent wide laminectomy and instrumentation fixation and fusion. (b) 65 y/o female, degenerative disc disease of L45, L5S1 with L345S1 spinal stenosis, who underwent wide laminectomy and instrumentation fixation and fusion.

All patients were placed in the prone position with a midline approach and underwent posterolateral fusion with an autogenous bone graft by wide laminectomy and segmental pedicle screw instrumentation with or without interbody fusion. The major indications of surgery were low back pain and claudication. The surgical levels were determined according to symptoms, physical examination, radiography, and MRI findings. All patients underwent wide decompressive laminectomy (Figure 1) surgery over the levels of spinal stenosis, meaning removal of the all the spinous process and the medial halves of both laminae, with excision of the ligamentum flavum above and below at the target levels [12] (Figure 2). The decision to use FloSeal and/or an ultrasonic bone scalpel was based on the timing of introduction of these two tools into our hospital. FloSeal was first introduced into our hospital in May 2013, and the ultrasonic bone scalpel followed in September 2014. Before FloSeal was introduced, we used electrosurgical units, such as unipolar or bipolar cautery, or bone wax, Surgicel, Gelfoam, or gauze packing, to manage intraoperative active bleeding or oozing in patients in group A. In patients in groups B and C, FloSeal was injected into the epidural space during the decompressive or interbody fusion procedure and onto decorticated bony surfaces and covered with moist saline-soaked gauze for a minimum of two minutes or at the end of the procedure. After hemostasis had been achieved, excess gelatine matrix not involved in formation of the clot was washed away gently from the epidural space with saline. The above steps were repeated if bleeding persisted.

Traditionally, we completed the decompressive procedure with a wide laminectomy using a Kerrison punch or osteotome for all patients in groups A and B. However, for patients in group C, the decompressive procedure began with removal of the spinous process and interspinous ligament complex using a rongeur, and a longitudinal laminar cut was made bilaterally at the lamina–facet junction, in addition to a longitudinal central cut over the base of the spinous process, using an ultrasonic bone scalpel after identification of the stenosis level. The device continued to penetrate the laminar bone and deepen the laminar cut until the underlying interior laminar cortex was penetrated and the ligamentum flavum was reached and exposed. The separated lamina bone was then removed using a combination of a hand rongeur and a Kerrison punch to resect the underlying ligamentum flavum. All pedicle screws were placed via identification of anatomic landmarks using the free-hand technique with intraoperative fluoroscopy assistance. In all patients who underwent spinal decompression surgery analyzed in this study, we routinely used a closed-wound suction drainage system (Hemovac drain) for removal of blood and extra fluid after surgery.

## 3. Results

### 3.1. Intraoperative Blood Loss

**Figure 3:**
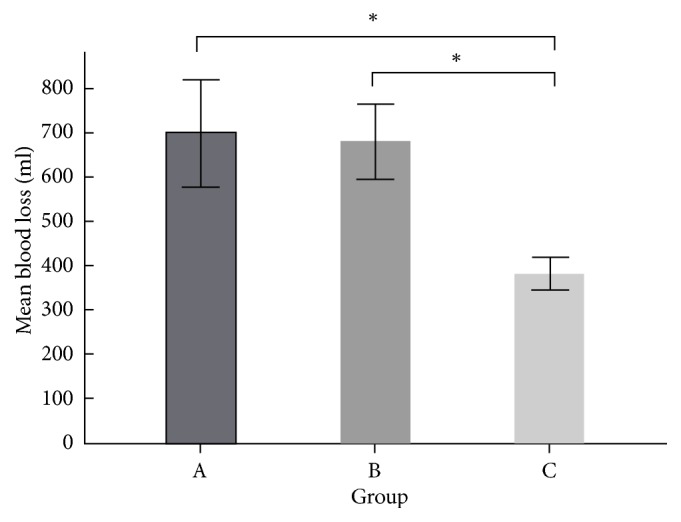
Mean intraop blood loss between groups. “*∗*” means* p* value <0.05.

A female predominance in each patient group was observed (group A, 54 (56%), group B, 72 (75%), and group C, 70 (69%)). The mean intraoperative blood loss was 700.2 ± 16.4 ml (range, 100–2500ml) in group A, 682.3 ± 13.2 ml (range, 150–2200 ml) in group B, and 383.2 ± 11.2 ml (range, 100–850 ml) in group C, with statistically significant differences (P < 0.05) between groups A and C and groups B and C. There was no statistical difference (P = 0.243) between groups A and B. With regard to subgroups, in the short-segment surgery subgroups, the mean intraoperative blood loss was 527.2 ± 12.2 ml in group A, 517.4 ± 12.2 ml in group B, and 266.3 ± 9.2 ml in group C, with the differences being statistically significant (P < 0.001) between groups A and C and groups B and C. In the long-segment surgery subgroups, the intraoperative blood loss was 1018.6 ± 25.3 ml in group A, 827.2 ± 15.1 ml in group B, and 463.2 ± 13.1 ml in group C, with statistically significant differences (P < 0.001) between groups A and C and groups B and C. No statistically significant difference (P = 0.474) was observed between groups A and B. The mean amount of FloSeal used in group B was 1.0 ± 0.2 packs (5 ± 1 ml) and in group C it was 1.3 ± 0.4 packs (6.5 l ± 2 ml) (Figure 3).

### 3.2. Surgical Duration

**Figure 4:**
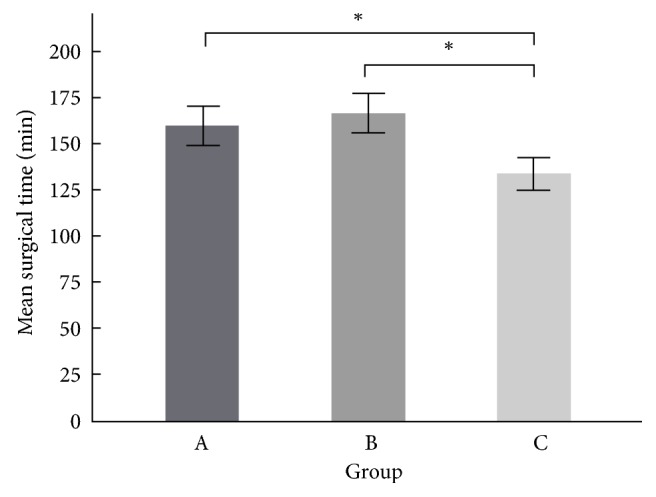
Mean surgical time between groups. “*∗*” means* p* value <0.05.

The mean surgical duration in group A was 160 ± 4.2 mins (range, 78~325 mins), in group B it was 167 ± 5.2 mins (range, 75–389 mins), and in group C it was 134 ± 2.5 mins (range, 32–222 mins). Statistically significant differences (P < 0.05) were observed between groups A and C and groups B and C, but not between groups A and B (P = 0.891). In the short-segment surgery subgroups, the mean surgical duration was 139 ± 2.1 mins in group A, 144 ± 2.8 mins in group B, and 101 ± 2.1 mins in group C, with statistically significant differences (P < 0.001) being observed between groups A and C and groups B and C, but not between groups A and B (P = 0.803). However, in the long-segment surgery subgroups, the mean surgical duration was 197 ± 3.2 mins in group A, 196 ± 3.6 mins in group B, and 169 ± 3.6 mins in group C, in which statistically significant differences (P < 0.001) were observed between groups A and C and groups B and C, but not between groups A and B (P = 0.991) (Figure 4).

### 3.3. Complications

In total, six patients included in this study sustained dura injuries during the decompressive procedure, three cases in group A, two in group B, and one in group C. All dura injuries were repaired immediately during surgery. Of these six patients, two in group A and one in group B suffered headaches and dizziness after surgery during their stay on the ward, and they recovered spontaneously after adequate hydration and rest without further neurologic deterioration. In the other three patients, no low-CSF syndrome or other neurologic deficits were observed after surgery. In the six patients, no CSF leakage from the wound was observed, and in no case was reoperation surgery required. None of the patients suffered from postoperative neurological deficits or symptomatic epidural hematoma.

## 4. Discussion

In recent years, numerous studies have identified that a prolonged surgical duration and significant perioperative blood loss are independent risk factors related to postoperative complications. Kim et al. [13] reported that an increased surgical duration was associated with stepwise increases in the risks of surgical complications and transfusions in patients who underwent single-level lumbar spine fusion surgery. Meanwhile, a prolonged anesthesia duration has also been found to be associated with increased risks of complications, such as VTE, an increased length of stay, and the need to return to the operating theatre [13–16]. Therefore, shortening the surgical duration and minimizing perioperative blood loss are major priorities in spinal surgery. One of the most critical steps in degenerative thoracolumbar spine surgery is the decompressive procedure. This procedure not only is the key step in terms of obtaining a satisfactory surgical outcome, but is also risky owing to the potential risks of dura or nerve injury, uncontrollable bleeding, and a lengthened surgical duration. Therefore, the ultrasonic bone scalpel was introduced for use in spinal surgery several years ago. Numerous studies have analyzed the safety of using an ultrasonic bone scalpel for bone-cutting in spinal surgery [2, 10]. Derya et al. reported a relatively short surgical duration and a lower need for blood replacement when using an ultrasonic bone shaver [17]. In our study, the surgical duration required for both short- and long-segment surgery differed significantly between groups A and C and groups B and C, being significantly shorter in group C than in groups A and B. In cases in group C, we used an ultrasonic bone scalpel for wide laminectomy at the target levels. In the patients in groups A and B, we performed the wide laminectomy using a Kerrison punch and rongeur at the target levels. The surgical duration did not differ significantly between groups A and B for both short- and long-segment surgery, though a longer surgical duration was noted for short-segment surgery in group B than in group A. The longer surgical duration in group B relative to group A may be related to the additional time required for FloSeal to work. According to the user guide for FloSeal, the median time to hemostasis is 90 seconds for every injection [11].

Intraoperative blood loss is a common problem, especially in multilevel spinal fusion procedures. Dafna et al. [18] reported the occurrence of significant blood loss leading to multiple blood transfusions in the intraoperative and postoperative periods during multilevel and even single-level spinal surgery. The rates of mortality and morbidity are prominently increased in patients who have a blood loss greater than 500 mL during spinal surgery [7]. As expected, the length of hospital stay for patients with substantial bleeding is prolonged concomitantly [7, 19]. An increased intraoperative blood loss also means an increased need for blood transfusions. Large blood transfusions increase not only the risks of metabolic and clotting abnormalities, but also electrolyte disturbances and disease transmission [18, 20]. Therefore, several pharmacologic agents and nonpharmacologic techniques have been developed to reduce intraoperative blood loss during major spinal surgery, one of which is the hemostatic gel matrix FloSeal. FloSeal has been reported not only to decrease the surgical duration and intraoperative blood loss in patients undergoing cardiac [21], general [22], and ENT [23] surgery, but also to decrease the intraoperative blood loss in corrective adolescent idiopathic scoliosis surgery [24]. Therefore, in this study, the intraoperative blood loss in group B was lower than that in group A in both short- and long-segment surgeries, but the difference was not statistically significant. The mean blood loss in group B (682.3 ± 13.2 ml) was also lower than that in group A (700.2 ± 16.4 ml), but the difference was not statistically significant. The mean amount of FloSeal used in group B was 1.0 ± 0.2 packs (5 ml/pack). Our data showed that the use of FloSeal assisted hemostasis in degenerative thoracolumbar spine surgery and appeared to have a positive effect in terms of reducing intraoperative blood loss. The amount of FloSeal used during surgery may play a key role in the quantity of blood lost. For economic reasons, we still employed conventional techniques for controlling bleeding, such as unipolar or bipolar cautery, bone wax, and Surgicel, in all patients in the three groups, with FloSeal mostly being used at the epidural space during unstoppable bleeding after the decompressive or interbody fusion procedure and on decorticated bony surfaces. On the other hand, the difference in intraoperative blood loss between groups B and C in both the short- and long-segment surgery subgroups was statistically significant, being lower in group C than in group B. The mean intraoperative blood loss was also significantly lower in group C (383.2 ± 11.2 ml) than in group B (682.3 ± 13.2 ml); P < 0.001. The ultrasonic bone scalpel has been reported to have a sealing effect over cut surfaces and requires less manipulation within the epidural space, thus limiting the overall blood loss by 30–40% as compared with standard osteotomes and rongeurs in corrective idiopathic scoliosis spine surgery [25].

Hu et al. [5] reported the ultrasonic bone scalpel not only to be safe, but also to perform as desired when used as a bone-cutting device to facilitate osteotomies in a variety of spinal surgeries. The major concerns related to the ultrasonic bone scalpel are thermal injury and incidental dural tearing intraoperatively. In this study, only one patient sustained an incidental dura tear after the decompressive procedure was performed using an ultrasonic bone scalpel in group C. The injured dura was immediately repaired with 8-0 Prolene after completion of the decompressive procedure, and neither further CSF leakage nor postoperative headaches or low-CSF syndromes were noted in this patient. Bydon et al. [2] and Hu et al. [5] reported the incidence of dura injury when using an ultrasonic bone scalpel in spinal surgery to be 1.5–5.7%. The incidence of dura injury was relatively lower in group C (1%) than in group A (3.1%) and group B (2%), in which conventional tools were used to complete the decompressive procedure. Neither postoperative neurologic deficits nor postoperative epidural hematomas were noted within the three groups.

This study had several potential limitations. It was a retrospective review study, and some factors such as perioperative blood pressure, level of interbody fusion, and the surgeon's skill level will have affected the volume of intraoperative blood loss and even the surgical duration. In addition, the findings reflect the experience of a single large medical center; hence, the results may not be representative of all patients undergoing thoracolumbar surgery in other institutes.

## 5. Conclusion

In recent decades, great strides have been made in promoting the quality of medical care, improving surgical safety, and reducing surgical complications by developing many new medications and tools, such as FloSeal and the ultrasonic bone scalpel for use in spinal surgery. In this first study to discuss the combined use of FloSeal and an ultrasonic bone scalpel in primary degenerative thoracolumbar spine surgery, the results indicated that this is an effective and safe technique that reduces intraoperative blood loss and shortens the surgical duration.

## Figures and Tables

**Table 1:** Demographics.

	Group A	Group B	Group C	*P* value
Gender (*M/F*)	42/54	24/72	31/70	0.018*∗*
Case number (*No.*)	96	96	101	0.918
Age (*year*)	65.3±7.1	68.7±6.3	65.2±6.5	0.179

OP level				

Short segment (*No.*)	62	54	53	0.215
Long segment (*No*.)	34	42	48	0.212

Mean surgical levels	3.2±0.6	3.5±0.7	3.4±0.6	0.962
(*No*.)				

OP assists				

FloSeal	-	**+**	**+**	
Bone Scalpel	-	-	+	

Mean number of used FloSeal (*pack*)	0	1.0±0.2	1.3±0.4	

Short segment was defined as decompression and instrumentation ≦ 3 levels and long segment was defined as decompression and instrumentation ≧ 4 levels.

**Table 2:** Mean intraop blood loss, mean surgical times, and complications in each group.

	Group 1	Group 2	Group 3	*P* value
Mean intraop	700.2±16.4	682.3±13.2	383.2±11.2	<0.001*∗*
Blood Loss (*ml*)				

Short segment (*ml*)	527.2±12.2	517.4±12.2	266.3±9.2	<0.001*∗*
Long segment (*ml*)	1018.6±25.3	827.2±15.1	463.2±13.1	<0.001*∗*

Mean surgical time (*min*)	160±4.2	167±5.2	134±2.5	<0.001*∗*

Short segment	139±2.1	144±2.8	101±2.1	<0.001*∗*
Long segment	197±3.2	196±3.6	169±3.6	<0.001*∗*

Complications				

Dura injury (*No.*)	3	2	1	
Postop neurological deficit (*No.*)	0	0	0	
Epidural hematoma (*No.*)	0	0	0	

## Data Availability

The retrospective data used to support the findings of this study are available from the corresponding author upon request.
