# Corrigendum to “Effects of Combined Use of Ultrasonic Bone Scalpel and Hemostatic Matrix on Perioperative Blood Loss and Surgical Duration in Degenerative Thoracolumbar Spine Surgery”

**DOI:** 10.1155/2019/2529153

**Published:** 2019-08-04

**Authors:** Hou-Tsung Chen, Chieh-Cheng Hsu, Meng-Lin Lu, Sung-Hsiung Chen, Jing-Miao Chen, Re-Wen Wu

**Affiliations:** ^1^Department of Orthopaedic Surgery, Kaohsiung Chang Gung Memorial Hospital, Kaohsiung, Taiwan; ^2^Graduate Institute of Clinical Medical Sciences, Chang Gung University, Taiwan

In the article titled “Effects of Combined Use of Ultrasonic Bone Scalpel and Hemostatic Matrix on Perioperative Blood Loss and Surgical Duration in Degenerative Thoracolumbar Spine Surgery” [1], the IRB number was given incorrectly as (IRB201700086B0). The correct number should be (IRB201600834B0). Therefore, the second sentence in subsection “2.1. Patients” should be updated as follows:

The study was conducted with a waiver of patient consent and was approved by the Institutional Review Board of our hospital (IRB201600834B0).
